# Noise Reduction with Recursive Filtering for More Accurate Parameter Identification of Electrochemical Sources and Interfaces †

**DOI:** 10.3390/s25123669

**Published:** 2025-06-11

**Authors:** Mitar Simić, Milan Medić, Milan Radovanović, Vladimir Risojević, Patricio Bulić

**Affiliations:** 1 Faculty of Electrical Engineering, University of Banja Luka, 78000 Banja Luka, Bosnia and Herzegovina; medicm.97@gmail.com (M.M.); vladimir.risojevic@etf.unibl.org (V.R.); 2 Faculty of Technical Sciences, University of Novi Sad, 21000 Novi Sad, Serbia; rmilan@uns.ac.rs; 3 Faculty of Computer and Information Science, University of Ljubljana, 1000 Ljubljana, Slovenia; patricio.bulic@fri.uni-lj.si

**Keywords:** battery impedance analysis, equivalent circuits, impedance analysis, parameter identification

## Abstract

Noise reduction is essential in analyzing electrochemical impedance spectroscopy (EIS) data for accurate parameter identification of models of electrochemical sources and interfaces. EIS is widely used to study the behavior of electrochemical systems as it provides information about the processes occurring at electrode surfaces. However, measurement noise can severely compromise the accuracy of parameter identification and the interpretation of EIS data. This paper presents methods for parameter identification of Randles (also known as R-RC or 2R-1C) equivalent electrical circuits and noise reduction in EIS data using recursive filtering. EIS data obtained at the estimated characteristic frequency is processed with three equations in the closed form for the parameter estimation of series resistance, charge transfer resistance, and double-layer capacitance. The proposed recursive filter enhances estimation accuracy in the presence of random noise. Filtering is embedded in the estimation procedure, while the optimal value of the recursive filter weighting factor is self-tuned based on the proposed search method. The distinguished feature is that the proposed method can process EIS data and perform estimation with filtering without any input from the user. Synthetic datasets and experimentally obtained impedance data of lithium-ion batteries were successfully processed using PC-based and microcontroller-based systems.

## 1. Introduction

**Figure 1:**
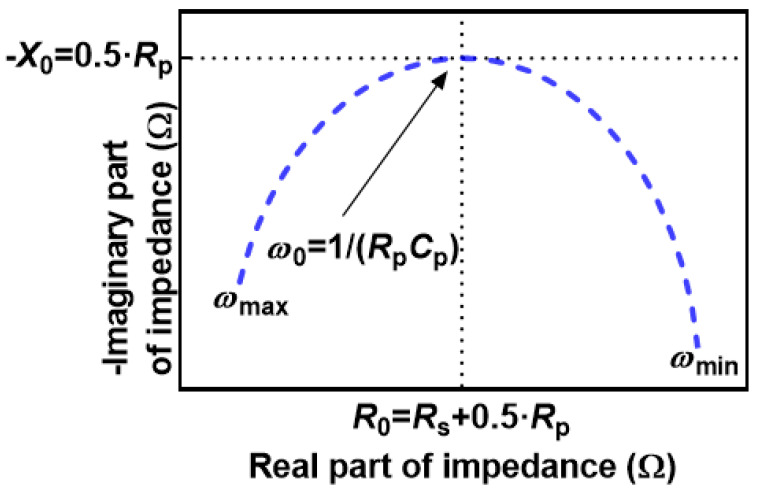
Nyquist plot of R-RC circuit and the corresponding values of *R*_0_, *X*_0_, and *ω*_0_. Please refer to the Section 2.2.

Electrical Impedance Spectroscopy (EIS) is a widely used method for characterizing electrochemical sources, such as lithium-ion batteries [1,2,3], photovoltaic elements [4], or general analysis of materials’ chemistry [5]. The noticeable feature of EIS is the possible correlation between the impedance of the analyzed element and its physical properties, even on the microstructural scale [6]. The full benefits of the EIS can be achieved with the proper processing of the measured impedance [7], and that task usually includes the modeling with the equivalent electrical circuits [8]. The structure and complexity of the appropriate equivalent electrical circuit to fit EIS data can help understate the specific object of interest. The first step in the EIS analysis is usually the creation of the Nyquist plot, which presents the real part of impedance (resistance) on the horizontal axis. In contrast, the negative-signed imaginary part (reactance) is plotted on the vertical axis, as shown later in Figure 1. For example, the presence of a single or higher number of relaxation processes can be identified by the semicircles on the Nyquist plot [9]. Relaxation processes are usually modeled with a parallel combination of resistor and capacitor (parallel RC circuit).

A commonly used equivalent electrical circuit is composed of two resistors and one capacitor, also known as the R-RC, 2R-1C, or simplified Randles circuit [10]. Among other applications [11], the R-RC circuit has been explored to monitor the corrosion of reinforced concrete [12], to study urea oxidation on nickel oxide nanoparticles modified glassy carbon [13], to model cement-based nanocomposites [14], to characterize intermetallic particles and the effect of chromate inhibitors [15], to study the cathodic hydrogen evolution reaction on polycrystalline rhenium [16], to investigate how electrical conduction occurs in a dielectric layer that is in contact with a mildly acidic solution [17], etc. Therefore, reliable and accurate parameter estimation of an R-RC circuit is of great importance for various fields of research and industry.

Classical methods for the parameter estimation of an R-RC circuit include PC-based software and non-linear least squares (NLLS) fitting. A wide span of EIS software for PC-based stations is available, such as Z-view, ZSimpWin, RelaxIS, Zman, EIS Spectrum Analyser, Multiple Electrochemical Impedance Spectra Parameterization (MEISP), etc. However, some of these solutions are not free of charge. Moreover, input data files must be formatted in a way that is compatible with the specific requirements of the software package used. Thus, such solutions are not optimized to be deployed on-site with convenient integration along with the measurement device in autonomous operation. On the other hand, the NLLS-based approaches allow a certain level of customization, but the main limitation is the requirement for the specific toolbox/function of the specific software, such as *lsqcurvefit* in MATLAB R2013b. NLLS-based approaches also require a very good set of initial values provided by the user. In addition, slow execution due to the need to simultaneously solve large sets of equations, as well as nonconvergence or convergence to the local minimum, are also reported [18].

As described above, EIS and parameter estimation of equivalent electrical circuits are powerful tools for analyzing electrochemical sources and interfaces. However, distorted impedance spectra due to the noise can lead to wrong interpretations and conclusions. Because of that, enhancing the signal quality (reduction in noise presence with respect to the useful signal) enables more reliable modeling of electrochemical systems, and it was the subject of work for many research groups [19]. For example, the use of pseudo-random sequences and multi-sine signals showed certain advantages in reducing noise and reducing the time of measurement [19]. Moreover, the optimization of frequency sets used in EIS measurements to reduce noise impact and improve the accuracy of equivalent circuit model parameter estimations is presented in [20]. The authors showed that by adjusting the selection of the frequency points, it is possible to achieve enhanced measurement efficiency without compromising data quality. Moreover, a discrete wavelet transform combined with multi-resolution analysis was shown to be capable of effectively denoising EIS data [21]. Finally, the recent works include the application of artificial intelligence and machine learning methods. For example, researchers developed a deep learning technique using artificial neural networks (ANNs) to identify equivalent circuit parameters from EIS data [22]. Similarly, a convolutional neural network (CNN) approach was proposed to pre-fit EIS spectra, generating initial parameters for complex non-linear least squares (CNLS) regression [23].

The primary objective of this study is to enhance a previously reported technique for identifying parameters in the R-RC circuit [24] with an embedded digital filter to reduce the impact of the measurement noise. The parameter extraction method relies on estimated values of the characteristic frequency, resistance, and reactance at that frequency. Notably, it operates independently of PC-based software and does not necessitate initial user-provided values. It is well-suited for implementation on embedded hardware, such as microcontroller-based boards. The key benefits include rapid estimation, as each parameter is governed by a single equation, facilitating the estimation of individual parameters. This characteristic is particularly valuable when dealing with time-dependent impedance caused by changes in a single parameter (physical process). However, as the proposed estimation method from [24] relies on the single-frequency impedance values, its accuracy might become affected by the measurement noise, and that presents the main motivation for this work.

## 2. Materials and Methods

### 2.1. The Structure and the Importance of the R-RC Equivalent Electrical Circuit for Electrochemistry

The R-RC circuit comprises a resistor *R*_s_ connected in series with a parallel combination of resistor *R*_p_ and capacitor *C*_p_. The complex impedance of an R-RC circuit *Z*(*jω*) can be broken down into two components: a real part denoted as *R*(*ω*) and an imaginary part denoted as *X*(*ω*).

(1) $$Z\left(j\omega \right)=R\left(\omega \right)+jX\left(\omega \right).$$

Both the real and imaginary components are dependent on the angular frequency *ω* (measured in radians per second) as well as the model parameters (*R*_s_, *R*_p_, and *C*_p_):

(2) $$R\left(\omega \right)=\frac{R_{s}+R_{p}+\omega^{2}{R_{s}R}_{p}^{2}C_{p}^{2}}{1+\omega^{2}R_{p}^{2}C_{p}^{2}},$$

(3) $$X\left(\omega \right)=\frac{-\omega C_{p}R_{p}^{2}}{1+\omega^{2}R_{p}^{2}C_{p}^{2}}.$$

In practical applications, measured values of *R* and *X* are available at a finite number of measurement points, *N*. The main task in EIS analysis and this work is to process these measurements and estimate values of model parameters (*R_s_*, *R_p_*, and *C_p_*).

Elements of the R-RC circuit (*R*_s_, *R*_p_, and *C*_p_) have physical interpretation, which enables a close correlation between the values of model parameters and the analyzed process [25]. Their interpretation might vary between the applications, but here, we will focus on the case of the battery impedance [26]. For instance, *R*_s_, also known as series resistance, stands for the battery’s internal resistance. Monitoring *R*_s_ can reveal the battery’s age and general condition. Parallel resistance (*R*_p_) is frequently associated with charge transport activities at the electrode–electrolyte interface. For instance, increased charge transfer resistance can be a sign of decreased electrode/electrolyte contact or deterioration of the electrode surface. *C*_p_ stands for the electrical double-layer capacitance at the electrode–electrolyte interface. It depicts the properties of the electrolyte, the accessibility and surface area of the electrode, and the double-layer structure. The accumulation of electrode/electrolyte interphases or the development of passivation layers are two examples of surface property changes that can be indicated by variations in *C*_p_.

### 2.2. The Method for Parameter Estimation of R-RC Equivalent Electrical Circuit [24]

Angular frequency, also known as characteristic angular frequency (*ω*_0_), is an angular frequency at which *X*(*ω*) has the minimum value. The analytical solution for the calculation of characteristic angular frequency is

(4) $$\omega_{0}=\frac{1}{R_{p}C_{p}}.$$

By substituting Equation (4) into (2) and (3), the respective values of the real and imaginary parts at this frequency can be calculated as follows:

(5) $$R_{0}=R\left(\omega_{0}\right)=R_{s}+\frac{R_{p}}{2},$$

(6) $$X_{0}=X\left(\omega_{0}\right)=-\frac{R_{p}}{2}.$$

The position of *R*_0_, *X*_0_, and *ω*_0_ on the Nyquist plot is given in Figure 1.

Analytical solution of Equations (4)–(6), with respect to model parameters (*R_s_*, *R_p_*, and *C_p_*), is given with the following equations [24]:

(7) $$R_{s}=R_{0}+X_{0},$$

(8) $$R_{p}=-2{\cdot X}_{0},$$

(9) $$C_{p}=-\frac{1}{2\cdot \omega_{0}{\cdot X}_{0}}.$$

Therefore, there is a very efficient method to estimate all three model parameters if the set of frequency points includes the *ω*_0_, or a value close to it.

The main advantages of the proposed estimation system are simplicity and computational efficiency, because only processing of the imaginary part of impedance for estimation of *ω*_0_ and *X*_0_ and estimation of *R*_0_ from the measured real part of impedance are needed. The estimation of *ω*_0_, *X*_0_, and *R*_0_ is followed by three calculations in total for three parameters (*R_s_*, *R_p_*, and *C_p_*). These tasks are not demanding in terms of computational resources, even for low-cost 8-bit microcontrollers. In addition to these features, the proposed system is free from the need for an initial guess or the requirement for a specific function or toolbox that is included in the software package. Such property adds extra value in terms of portability and very convenient deployment on another hardware platform. The distinguished feature is the ability to estimate the value of just one parameter of interest (any of *R*_s_, *R*_p_ or *C*_p_) without needing to solve the complete set of equations for all three parameters. This can be very useful when the specific process is monitored.

However, as the proposed estimation method from [24] relies on the single-frequency impedance values, its accuracy might be affected by the measurement noise, as demonstrated in Section 3. The main goal of the work presented here is to improve noise immunity of the estimation method without significant degradation of performance in terms of complexity and processing time.

### 2.3. Impact of Limited Number of Measurement Points on the Estimation Accuracy

The inclusion of the characteristic frequency as the point on which measurement will be performed is of crucial importance for the proposed method. However, the limited number of measurement points leads to the very reasonable expectation that exact values might not be included in the measurement. That means that instead of the exact value of *ω*_0_, a close value of α∙*ω*_0_ might be included, where α is a real number that indicates an error in characteristic frequency estimation. For example, α = 0.95 and α = 1.05 are for a relative error of ±5% in characteristic frequency estimation.

Therefore, instead of *R*_0_ and *X*_0_ given with (5) and (6), respectively, the values of real and imaginary parts of impedance at the characteristic frequency become

(10) $${R^{\prime }}_{0}=R\left(\alpha \cdot \omega_{0}\right)=R_{s}+\frac{R_{p}}{1+\alpha^{2}},$$

(11) $${X^{\prime }}_{0}=X\left(\alpha \cdot \omega_{0}\right)=-\frac{\alpha \cdot R_{p}}{1+\alpha^{2}}.$$

The left sides of (10) and (11) represent measured values at the estimated resonant frequency α∙*ω*_0_.

An analytical solution for model parameters, which includes error in characteristic resonant frequency and the corresponding resistance and reactance at that frequency, is

(12) $${R^{\prime }}_{s}={R^{\prime }}_{0}+\frac{{X^{\prime }}_{0}}{\alpha },$$

(13) $${R^{\prime }}_{p}=-\frac{(1+\alpha^{2})\cdot {X^{\prime }}_{0}}{\alpha },$$

(14) $${C^{\prime }}_{p}=-\frac{1}{2\cdot \alpha \cdot \omega_{0}\cdot {X^{\prime }}_{0}}.$$

Set of Equations (12)–(14) could be used if α is known, and in that case, the estimation of the exact values of model parameters would be possible. Therefore, by analyzing the sets of Equations (12)–(14), it can be observed that the described estimation method is sensitive to the errors in characteristic frequency estimation, as well as the corresponding values of real and imaginary parts of impedance at that frequency.

The approximate value of α can be determined only in the case of an R-RC circuit made from discrete components with very small tolerances in values of used elements. However, such circuits are of low interest for practical applications, as commonly analyzed electrochemical solutions are characterized by EIS measurements without reference values of model parameters.

However, the maximum value of α can be determined with relatively high accuracy by following the analysis. Frequency sweep is performed from *ω*_min_ to *ω*_max_ with a certain step Δ*ω*. The actual characteristic frequency *ω*_0_ lies between two points (*ω*_i_ and *ω*_i+1_, for example). Relative error will be the biggest if *ω*_0_ is in the middle between two frequency points. Otherwise, it will be smaller, as it will be rounded and estimated either as *ω*_i_ or *ω*_i+1_.

Therefore, α_max_ can be calculated as follows:

(15) $$\alpha_{max}=\frac{\omega_{i}+\Delta \omega }{\omega_{0}}=\frac{\omega_{i}+(\omega_{i+1}-\omega_{i})}{\omega_{0}}=\frac{\omega_{i+1}}{\omega_{0}}.$$

α_max_ will be smaller if the characteristic frequency *ω*_0_ is higher when compared to *ω*_i+1_ and Δ*ω*. For example, the frequency range is from *ω*_min_ = π krad/s to *ω*_max_ = 100π krad/s with the linear step Δω = π krad/s. If the characteristic angular frequency is equal to *ω*_0_ = 1.5π krad/s, using (15), a value of α = 2/1.5 ≈ 1.33 is calculated. On the other hand, if *ω*_0_ = 91.5π krad/s, using (15), a value of α = 92/91.5 ≈ 1.0055 is calculated. The actual value of α will be smaller if *ω*_0_ is closer to one of the frequency points. However, it is only possible to determine the maximum possible value of α by using (15) and by setting $\omega_{0}$ to be equal to the minimal angular frequency in the measurement range.

Therefore, a possible solution for the higher estimation accuracy is a smaller frequency step, but that will lead to a higher number of measurement points. Consequently, the complexity and price of the measurement and data acquisition device are increased. Another solution is to use logarithmic frequency distribution because that will ensure the frequency-independent value of *α*_max_. However, *α*_max_ will be higher at higher frequencies in the case of the logarithmic distribution when compared to the linear frequency distribution. In the case of linear frequency distribution, it will become smaller at the higher frequencies [refer to Equation (15)]. On the other hand, with the same number of measurement points, the frequency step Δ*ω* will be bigger at higher frequencies in the case of logarithmic distribution.

### 2.4. Impact of Noise on the Estimation Accuracy

Even if the actual value of characteristic frequency is included in the dataset, measured values of real and imaginary parts can be inaccurate because of the measurement noise. Noise can be caused by the non-ideal properties of the measurement device, electromagnetic interference, human error, etc. The impact of the noise on the estimation accuracy can be analyzed by finding the sensitivities *S* of equations for estimated values of model parameters to changes in values of *R*_0_ and *X*_0_,

(16) $$S_{R_{0}}^{R_{s}}+S_{X_{0}}^{R_{s}}=\frac{R_{0}}{R_{s}}\frac{\partial R_{s}}{\partial R_{0}}+\frac{X_{0}}{R_{s}}\frac{\partial R_{s}}{\partial X_{0}}=\frac{R_{0}}{R_{0}+X_{0}}+\frac{X_{0}}{R_{0}+X_{0}}=1,$$

(17) $$S_{R_{0}}^{R_{p}}+S_{X_{0}}^{R_{p}}=\frac{R_{0}}{R_{p}}\frac{\partial R_{p}}{\partial R_{0}}+\frac{X_{0}}{R_{p}}\frac{\partial R_{p}}{\partial X_{0}}=-2\frac{X_{0}}{R_{p}}=1,$$

(18) $$S_{R_{0}}^{C_{p}}+S_{X_{0}}^{C_{p}}=\frac{R_{0}}{C_{p}}\frac{\partial C_{p}}{\partial R_{0}}+\frac{X_{0}}{C_{p}}\frac{\partial C_{p}}{\partial X_{0}}=\frac{R_{p}}{2X_{0}}=-1,$$

where $S_{R_{0}}^{R_{s}}$ denotes the sensitivity of parameters *R*_s_ to small variations in the value of *R*_0_, and so on.

It can be observed from (16)–(18) that space for reducing the sensitivities of model parameters to small variations of *R*_0_ and *X*_0_ is very limited, as sensitivities of *R*_s_, *R*_p_ and *C*_p_ are constant and directly proportional to small variations of *R*_0_ and *X*_0_. Therefore, the possible solution is signal filtering, which is proposed in this paper by the method described in the following Section 2.5.

### 2.5. Recursive Filtering and the Selection of the Weighting Factor w

The exponential filter belongs to the group of recursive filters. A filtered value is calculated as the sum of the new measurement multiplied by the weighting factor *w* and the previous filtered value multiplied by (1 − *w*). This procedure applied on measured resistance *R* and reactance *X* gives filtered resistance and reactance ($\tilde{R}$ and $\tilde{X}$),

(19) $${\tilde{R}}_{i}=w\cdot R_{i}+(1-w)\cdot {\tilde{R}}_{i-1},$$

(20) $${\tilde{X}}_{i}=w\cdot X_{i}+(1-w)\cdot {\tilde{X}}_{i-1},$$

where *w* is in the range [0, 1]. The selection of the weighting factor *w* value might be very challenging because it requires a balance between the fast response and the appropriate level of smoothing.

There is no strict rule on how to select a value for *w*. However, the main purpose of filtering is to reduce noise because it causes deviations of impedance from the expected form, described in (2) and (3) in the case of the electrochemical element that can be modeled with the Randles circuit. Because of that, our proposed method integrates filtering into the estimation procedure and selects the value of the weighting factor based on the minimum difference between measured and estimated values of impedance. Raw data of both arrays are first filtered with *w* = 0. Filtering is followed by the estimation of model parameters using the selected estimation method and filtered data (instead of measured). Calculation of real and imaginary parts of impedance (*R_est_*, *X_est_*) using the estimated values of model parameters is then performed using (2) and (3). Finally, the Root Mean Squared Error (RMSE) between estimated and measured data is calculated. The procedure above is performed for the next value of *w* in the range [0, 1], which is defined with step Δ*w* = 0.01. Value for Δ*w* can be changed, but we found that 0.01 works very well as the compromise between the accuracy and the computational time. The optimal value for *w* is selected based on the minimum RMSE sum for measured resistance and reactance (*R* and *X*) and estimated resistance and reactance (*R*_est_ and *X*_est_).

(21) $$\underset{w}{\mathrm{argmin}}\left\{\sqrt{\frac{1}{N}\sum_{i=0}^{N}{\left(R_{est}\left({w,\;\omega }_{i}\right)-R\left(\omega_{i}\right)\right)}^{2}}+\sqrt{\frac{1}{N}\sum_{i=0}^{N}{\left(X_{est}\left({w,\;\omega }_{i}\right)-X\left(\omega_{i}\right)\right)}^{2}}\right\}.$$

## 3. Simulation Results

### 3.1. The Synthetic Datasets

**Figure 2:**
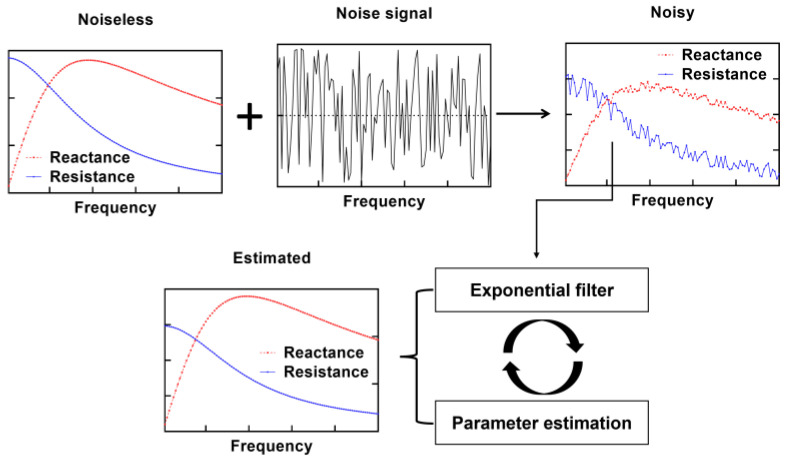
The steps in the generation and processing of synthetic impedance datasets with the proposed method.

We utilized Equations (2) and (3) to generate synthetic values for the real and imaginary components of impedance using the randomly selected nominal values of model parameters. The noise level was controlled using the function for random number generation, *rng*. A general-purpose pseudorandom number generator, *Mersenne Twister,* was specified. Noisy impedance was filtered using the proposed method followed by the estimation without reference to the idealistic values (noiseless), as shown in Figure 2. The analysis based on the synthetic datasets was used to determine the impact of frequency distribution type (Section 3.2), noise impact on the estimation accuracy (Section 3.3), analysis of characteristic frequency impact (Section 3.4), to demonstrate how recursive filtering reduces the noise impact and how the estimation accuracy of previously published work is enhanced with filtering (Section 3.5), and reduced number of data points (Section 3.6).

### 3.2. Impact of Frequency Distribution Type

Firstly, we created a synthetic real and imaginary part of impedance with the nominal values of model parameters (*R_s_* = 330 Ω, *R_p_* = 750 Ω and *C_p_* = 10 nF). These values were randomly chosen from a set of commercially available nominal values for discrete components. We conducted measurements at a total of *N* = 100 data points, spanning a frequency range from 1 kHz to 100 kHz with linear and logarithmic distribution. The summary of the estimated values and the corresponding relative errors (RE) is given in Table 1. It can be noticed that the proposed method is capable of estimating the values of model parameters with both frequency distribution types. In this analyzed case, slightly higher errors were observed in the case of the logarithmic distribution.

As discussed in Section 2.3, the relative errors will be lower if the ratio of the frequency step and actual characteristic frequency [given with Equation (15)] is smaller. To validate this assumption, another set of model parameters was used (*R*_s_ = 330 Ω, *R*_p_ = 750 Ω and *C*_p_ = 4.7 nF), which should correspond to the higher value of the characteristic frequency (≈45 kHz > ≈21 kHz). The results obtained (Table 1) support our analysis, which is given in Section 2.3.

### 3.3. Noise Impact on the Estimation Accuracy

Instead of ideal values of the real and imaginary parts of impedance, which were used in Section 3.2, random noise with levels of 0.5%, 1%, 5% and 10% was also added to *R* and *X* with the aim of investigating the noise impact on the estimation accuracy. We created 1000 datasets of impedance spectra calculated with nominal parameters of *R_s_* = 220 Ω, *R_p_* = 1 kΩ and *C_p_* = 3.3 nF (as reported in [24]), and the noise was randomly generated for each dataset. So, in total, we performed 4000 tests (1000 per noise level) at *N* = 100 data points, spanning a frequency range from 1 kHz to 100 kHz with linear distribution [24]. Estimated values of model parameters are then processed to determine mean and standard deviation values, as summarized in Table 2. It is evident that there are higher deviations of estimated values from the nominal values and the corresponding standard deviations with increasing noise level. These results present the main motivation for our work of denoising the EIS data (Section 3.5).

### 3.4. Analysis of Characteristic Frequency Impact

Based on the discussion given in Section 2.4, it was interesting to analyze datasets with the different values of characteristic frequency *f*_0_ and the corresponding values of *R*_0_ and *X*_0_. The dataset with nominal values of model parameters as follows: *R_s_* = 220 Ω, *R_p_* = 470 Ω and *C_p_* = 22 nF, and varying noise levels from 0.5% to 1%, was first processed. The frequency range and number of data points were the same as in Section 3.3. The corresponding values at characteristic frequency are *f*_0_ = 15,392.16 Hz, *R*_0_ = 455.00 Ω and *X*_0_ = −235.00 Ω. The summary of the estimated values and the corresponding relative errors (RE) is given in Table 3. A dataset with nominal model parameter values of *R_s_* = 1000 Ω, *R_p_* = 220 Ω and *C_p_* = 22 nF was also processed. The corresponding values at characteristic frequency are *f*_0_ = 32,883.25 Hz, *R*_0_ = 1110.00 Ω and *X*_0_ = −110.00 Ω. The summary of the estimated values and the corresponding relative errors is also given in Table 3.

To provide a more quantitative analysis, we performed a simulation analysis with 10 randomly generated values of nominal parameters and the corresponding values of characteristic frequency *f*_0_ (10 Ω < *R*_s_, *R*_p_ < 1000 Ω, 1 nF < *C*_p_ < 100 nF, 1 kHz < *f*_0_ < 100 kHz). The parameters of the frequency range from 1 kHz to 100 kHz and the number of data points *N* = 100 with linear distribution were not changed. It can be seen from Table 4 that when relative errors in characteristic frequency estimation are small, for example, for datasets 8 and 10, relative errors in estimated values of *R*_s_, *R*_p_ and *C*_p_ are smaller when compared to datasets with higher error in *f*_0_ estimation, for example, datasets 5 and 9.

### 3.5. Recursive Filtering for Noise Impact Reduction and Comparison with Previous Work

**Figure 3:**
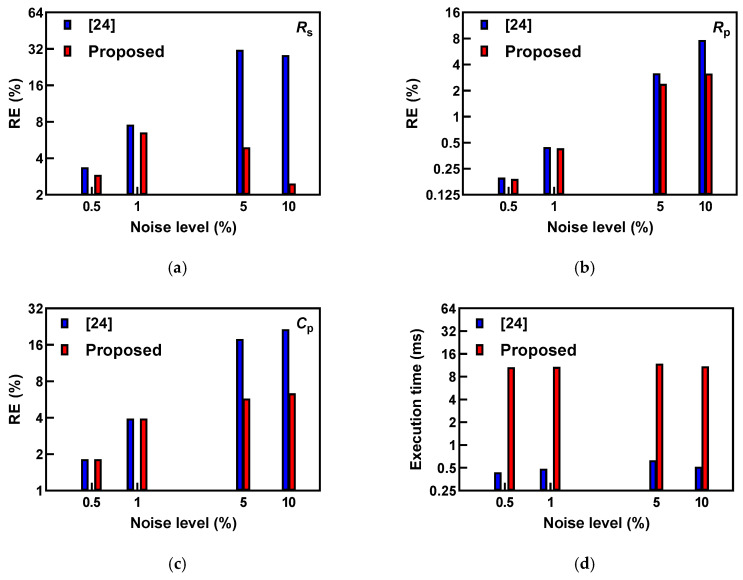
(**a**–**c**) Comparison of the relative errors for two estimation methods: raw data as presented in [24] and the proposed method in this work, and (**d**) comparison of execution times.

We first performed the comparison of the EIS data processing method presented in [24]. The dataset was created using the nominal values of model parameters (*R*_s_ = 220 Ω, *R*_p_ = 1 kΩ and *C*_p_ = 3.3 nF) and a frequency range from 1 kHz to 100 kHz (number of data points *N* = 100, with linear spacing). A comparison of the estimated values with the proposed method and previously reported [24] is summarized in Figure 3. Please note that the logarithmic scale was used on vertical axes in Figure 3. The proposed method in this work reduced the relative errors in estimated values for all noise levels. The benefits of the proposed method are evident, especially in cases with increased noise levels (5% and 10%). We also observed that estimation time (measured using built-in functions *tic* and *toc*) for both methods was consistent through the variations in noise levels, but that the proposed filtering increased estimation time by approximately 10 ms.

We then used the same impedance datasets from Section 3.2 with varying noise levels and processed them with the proposed integrated method of filtering and parameter estimation. Estimated values of model parameters for each dataset are then processed to determine mean and standard deviation values of the filter coefficient and parameters of equivalent electrical circuits, as summarized in Table 5. It is evident that there are higher deviations of estimated values from the nominal values and the corresponding standard deviations with increasing noise level. Moreover, the total RMSE (sum of RMSE for real and imaginary parts of impedance) is also improved when compared to the estimated values without the proposed filtering (>8%). These results confirmed the main objective of this work and the positive effects of denoising the EIS data for parameter estimation of equivalent electrical circuits (Section 3.2).

### 3.6. Reduced Number of Frequency Points

A reduced number of frequency points is also a very interesting subject for analysis, as it might have a direct impact on the estimation of characteristic frequency *f*_0_ (Section 3.4). A synthetic dataset was created with nominal values of model parameters (*R*_s_ = 330 Ω, *R*_p_ = 100 Ω and *C*_p_ = 51.34 nF) in such a way that the characteristic frequency is equal to 31 kHz. The frequency range 1 kHz–100 kHz was covered with linear spacing by different numbers of *N*, starting with 200, 150, 100, 50 and finally 20. The noise level also varied from 0% to 0.5% and finally 1%.

**Figure 4:**
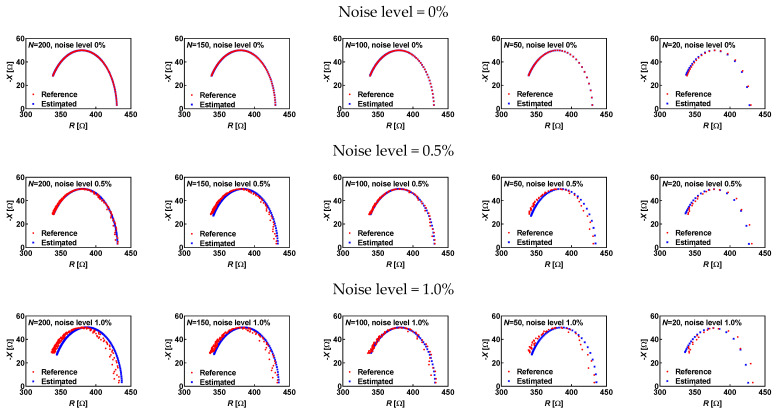
Nyquist plots of reference (red circles) and estimated (blue crosses) values in the case of different noise levels (0%, 0.5% and 1%) and different numbers of frequency points (*N* = 200, 150, 100, 50, 20).

In the case of noiseless data (noise level 0%), estimated values in cases of *N* equal to 200, 100, and 50 are equal to the reference values. This was expected because there was no noise, and the actual characteristic frequency matched the frequency points in the dataset. However, that condition was not satisfied in cases of *N* = 150 and *N* = 20. Relative errors in characteristic frequency estimation equal 0.33% for *N* = 150 and 4.07% for *N* = 20. The impact of the higher error in the estimation of characteristic frequency can be seen in higher relative errors (0.59%, 0.08%, and 3.84%) for the estimation of model parameters (*R*_s_ = 328.04 Ω, *R*_p_ = 99.92 Ω and *C*_p_ = 49.37 nF) in the case of *N* = 20 when compared to relative errors (0.05%, 0.00% and 0.33%) and estimated values of model parameters (*R*_s_ = 330.16 Ω, *R*_p_ = 100.00 Ω and *C*_p_ = 51.51 nF) in the case of *N* = 150. The corresponding Nyquist plots are shown in Figure 4.

A comparison of the estimated values for different numbers of measurement points and noise levels is summarized in Table 6. With *N* = 100, the characteristic frequency was estimated without error for all noise levels. However, the increased noise level has an impact on the estimated values of model parameters because of deviations in *R*_0_ and *X*_0_.

## 4. Experimental Results

### 4.1. Experimentally Obtained Impedance of Li-Ion Battery: Comparison with the PC-Based Software and the Related Works

We used a part (12 points in the frequency range of 0.3372 Hz to 8 Hz) of EIS measurement labeled as ID = 3541 within the publicly available dataset of a 2.9 Ah Panasonic 18650PF cell [27]. The Digatron Firing Circuits Universal Battery Tester was used for measurements inside a thermal chamber (temperature was kept constant at 25 °C). Estimated values of model parameters are *R*_s_ = 27.13 Ω, *R*_p_ = 28.54 Ω and *C*_p_ = 3.93 mF with filtering coefficient *w* = 0.92. The corresponding root mean square errors (RMSEs) for the real and imaginary parts of impedance are 0.47 Ω and 1.02 Ω, respectively, and the sum is lower than in the case of the original method [24], as shown in Table 7.

The same battery impedance dataset was used for parameter estimation using the PC-based software MEISP 3.0. The estimated values of model parameters are *R*_s_ = 26.58 Ω, *R*_p_ = 29.07 Ω and *C*_p_ = 3.56 mF. In addition, model parameter values are estimated using the two reported works in [28,29], as summarized in Table 7. A method presented in [28] uses the measured real and imaginary parts of impedance and the estimated value of characteristic frequency, with the solutions for three parameters found as the average of estimation on a complete set of frequencies, while work presented in [29] uses the measured real and imaginary parts of impedance, as well as the numerical approximation of the first derivative of the real/imaginary part quotient, followed by the averaging of values estimated on a complete set of frequencies. The method from [24] uses only values of characteristic frequency with real and imaginary parts of impedance at the frequency, which is a significantly smaller dataset, as it processes impedance values from the single frequency. However, all three methods [24,28,29] and commercial software used as a baseline in this research (MEISP) have one limitation in common: lack of data filtering embedded into the estimation procedure, which is the novelty of the work presented in this paper. A comparison of RMSEs for estimated resistance (RMSE*_R_*) and reactance (RMSE*_X_*) is also given. RMSE*_R_* and RMSE*_X_* were calculated using the following equations:

(22) $${RMSE}_{R}=\sqrt{\frac{1}{N}\sum_{i=0}^{N}{\left(R_{est}\left({w,\;\omega }_{i}\right)-R\left(\omega_{i}\right)\right)}^{2}},$$

(23) $${RMSE}_{X}=\sqrt{\frac{1}{N}\sum_{i=0}^{N}{\left(X_{est}\left({w,\;\omega }_{i}\right)-X\left(\omega_{i}\right)\right)}^{2}}$$

where *R*_est_/*X*_est_ are estimated resistance/reactance and *R*/*X* are measured resistance/reactance.

**Figure 5:**
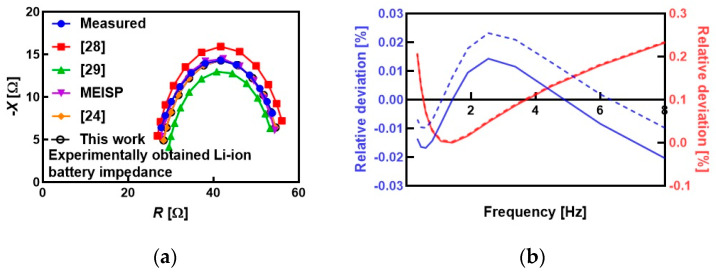
(**a**) Comparison of Nyquist plots for different fitting approaches [24,28,29], (**b**) comparison of relative deviations between the raw (solid) and estimated with the proposed method (dashed) resistance (blue colored) and reactance (red colored).

The Nyquist plots of measured impedance and estimated impedance with different approaches are shown in Figure 5a. The comparison of relative deviations between the measured and estimated values of resistance and reactance for the previously reported estimation method and the proposed method is shown in Figure 5b, indicating the improvement after filtering.

Table 7 presents a comparative analysis of the estimation results obtained using the proposed method and three existing approaches [24,28,29]. The proposed method yields the lowest RMSE for resistance and reactance (0.47 Ω and 1.02 Ω) when compared to previously published methods (0.54 Ω and 1.01 Ω) [24], (1.46 Ω and 0.96 Ω) [28], and (0.80 Ω and 3.50 Ω) [29], indicating a significant improvement and quantitatively confirming the enhanced estimation precision achieved by the proposed approach.

The estimation method proposed in this paper cannot compete with the accuracy of the PC-based EIS software (MEISP) in terms of RMSE. Still, the significant advantage is that our approach is platform-independent and does not require a specific operating system or user to manipulate the software during the estimation procedure. In addition to that, our estimation set of Equations (7)–(9) is independent, without cross-sensitivity among parameters, which means that if needed, just a subset of parameters can be estimated. For example, if some application requires just monitoring of *R*_p_, which is very often related to the charge transfer resistance, there is no need to estimate the full set of parameters. That is not possible with MEISP or other non-linear least squares approaches. On the other hand, the proposed work is more accurate here than the other two related works [28,29].

It also should be noted that the proposed fitting accuracy of <0.22% [Figure 5b] is close to the commercial MEISP software (please refer to Table 7), but it is also comparable to the values reported in the literature for real-life applications, such as less than 0.3% in the case of SoC estimation on Li-ion batteries [30], less than 5% [31] for online SOC/SOH estimation, from 0.11% to 3.34% [32] in the case of Li-ion battery modeling, and from 0.85% to 4.72% across different battery chemistries and conditions with a focus on battery health assessment [33].

### 4.2. Microcontroller-Based Implementation

We used the Arduino Nano Every board (Arduino, Monza, Italy) to deploy the estimation method on the microcontroller. The Arduino Nano Every board is based on the ATMega4809 (48 kB of flash memory, 6 kB of RAM and a clock speed of 20 MHz). Overall dimensions of 45 mm × 18 mm make it a preferable choice for portable devices.

The dataset was created in MATLAB R2013b using the nominal values of model parameters (noise level was 1.0%) and imported into the microcontroller’s program memory. The frequency range from 1 kHz to 100 kHz was linearly spaced with *N* = 100. Estimated values are the same as with MATLAB implementation (please refer to Table 3): *R*_s_ = 205.62 Ω, *R*_p_ = 1004.30 Ω and *C*_p_ = 3.17 nF. The optimal filtering coefficient was also the same: *w* = 0.81. The noticeable result is the execution time of just 2.79 s, which is needed for finding the optimal filter coefficient and parameter estimation using the filtered data. Moreover, the program code uses 16% of program storage space (8206/49,152 bytes), while the global variables use 23% of dynamic memory, leaving 4723 bytes for local variables. The program code is available on this link [34].

Here, it should be noted that achieving the same results with both MATLAB and the Arduino IDE 1.8.5 demonstrates that the proposed signal processing can be accurately implemented on resource-constrained embedded systems. This validates the mathematical integrity of the algorithm across platforms and proves that real-time signal processing and parameter extraction are feasible on low-power microcontrollers without sacrificing precision.

## 5. Conclusions and Future Work

The primary objective of this study was to develop an integrated method composed of the analytical solution for parameter estimation of the R-RC model (comprising three closed-form equations) with enhanced immunity to the measurement noise because of implemented digital filtering. A comprehensive analysis of the proposed system in terms of the estimation accuracy and impact of the limited number of data points in impedance spectra, as well as measurement noise, showed that the proposed method could provide accurate and reliable estimation results.

The key advantage of the proposed method is that it simplifies the estimation process by requiring only the values of the characteristic frequency, resistance, and reactance for accurate parameter estimation, as well as noise impact reduction with auto-tuned filtering. The simplicity and low complexity of the derived equations enable reliable deployment on the microcontroller-based board without an operating system. The lack of need for a specific toolbox or software package provides a great level of portability in terms of deployment on other hardware. The main limitation of the proposed work is that it relies on the accuracy of the characteristic frequency estimation and the corresponding real and imaginary parts of that frequency. Therefore, the set of measurement frequencies must include frequencies close to the value of the characteristic frequency. That also means that if the frequency range is, for example, from 1 kHz to 100 kHz, the range of values of model parameters can typically cover two decades, as we demonstrated in the simulation study in Section 3.4 (10 Ω < *R*_s_, *R*_p_ < 1000 Ω and 1 nF < *C*_p_ < 100 nF).

Our future work will include processing impedance datasets obtained for different electrochemical sources under different operating and environmental conditions.

## Figures and Tables

**Table 1:** The comparison of the estimated values and the corresponding relative errors for different frequency distribution types (linear or logarithmic).

Reference Values: *R_s_* = 330 Ω, *R_p_* = 750 Ω. *C_p_* = 10 nF and *f*_0_ = 21,220.66 Hz										
Frequency Distribution Type	Frequency Range	Number of Points	*R_s_* [Ω]	RE [%]	*R_p_* [Ω]	RE [%]	*C_p_* [nF]	RE [%]	*f*_0_ [Hz]	RE [%]
Linear	1 kHz–100 kHz	100	333.94	1.19	749.96	0.01	10.11	1.06	21,000.00	1.04
Logarithmic			324.37	1.71	749.91	0.01	9.85	1.49	21,544.35	1.53
**Reference Values: *R_s_* = 330 Ω, *R_p_* = 750 Ω. *C_p_* = 4.7 nF and *f*_0_ = 45,150.34 Hz**										
**Frequency Distribution Type**	**Frequency Range**	**Number of Points**	***R_s_* [Ω]**	**RE [%]**	***R_p_* [Ω]**	**RE [%]**	***C_p_* [nF]**	**RE [%]**	***f*_0_ [Hz]**	**RE [%]**
Linear	1 kHz–100 kHz	100	331.25	0.38	750.00	<0.005	4.72	0.33	45,000.00	0.33
Logarithmic			328.36	0.50	749.99	<0.005	4.68	0.44	45,348.79	0.44

**Table 2:** The comparison of the mean ± standard deviations of estimated values for 1000 randomly generated datasets with different noise levels.

Noise Level [%]	*R_s_* [Ω]	*R_p_* [Ω]	*C_p_* [nF]
	220 Ω	1000 Ω	3.3 nF
0.5%	221.03 ± 17.02	1003.10 ± 1.24	3.29 ± 0.112
1.0%	221.26 ± 22.21	1006.96 ± 2.06	3.27 ± 0.145
5.0%	228.54 ± 39.67	1040.94 ± 6.17	3.16 ± 0.243
10.0%	237.54 ± 50.09	1085.33 ± 10.01	3.03 ± 0.285

**Table 3:** The comparison of the estimated values and the corresponding relative errors for different frequency distribution types and two noise levels.

Reference Values: *R_s_* = 220 Ω, *R_p_* = 470 Ω. *C_p_* = 22 nF and *f*_0_ = 15,392.16 Hz											
Frequency Distribution Type	Frequency Range	Noise Level	Number of Points	*R_s_* [Ω]	RE [%]	*R_p_* [Ω]	RE [%]	*C_p_* [nF]	RE [%]	*f*_0_ [Hz]	RE [%]
Linear	1 kHz–100 kHz	0.5%	100	226.82	3.10	471.25	0.27	22.52	2.34	15,000.00	2.55
Logarithmic				207.49	5.69	470.41	0.09	20.76	5.64	16,297.51	5.88
Linear		1%		227.50	3.41	472.67	0.57	22.45	2.04	15,000.00	2.55
Logarithmic				208.01	5.45	471.59	0.34	20.71	5.87	16,297.51	5.88
**Reference Values: *R_s_* = 1000 Ω, *R_p_* = 220 Ω. *C_p_* = 22 nF and *f*_0_ = 32,883.25 Hz**											
**Frequency Distribution Type**	**Frequency Range**	**Noise Level**	**Number of Points**	***R_s_* [Ω]**	**RE [%]**	***R_p_* [Ω]**	**RE [%]**	***C_p_* [nF]**	**RE [%]**	***f*_0_ [Hz]**	**RE [%]**
Linear	1 kHz–100 kHz	0.5%	100	1008.75	0.87	220.07	0.03	23.33	6.04	31,000.00	5.73
Logarithmic				1010.03	1.00	220.66	0.30	23.08	4.89	31,257.16	4.95
Linear		1%		1010.82	1.08	220.52	0.24	23.28	5.82	31,000.00	5.73
Logarithmic				1014.35	1.43	221.60	0.73	22.98	4.44	31,257.16	4.95

**Table 4:** The comparison of the estimated values and the corresponding relative errors for different values of characteristic frequency *f*_0_ within the range from 1 kHz to 100 kHz.

Dataset No.	*f*_0_ [kHz]		RE [%]	*R_s_* [Ω]		RE [%]	*R_p_* [Ω]		RE [%]	*C_p_* [nF]		RE [%]
	Reference	Estimated		Reference	Estimated		Reference	Estimated		Reference	Estimated	
1	61.88	62.00	0.19	346.79	346.60	0.06	334.20	334.20	<0.005	7.69	7.68	0.12
2	29.74	30.00	0.87	884.24	883.66	0.07	130.70	130.70	<0.005	40.95	40.59	0.88
3	75.71	76.00	0.38	798.75	797.73	0.13	534.33	534.33	<0.005	3.93	3.91	0.51
4	25.17	25.00	−0.68	63.67	64.60	1.46	267.10	267.09	<0.005	23.67	23.84	0.72
5	66.50	66.00	−0.75	617.70	621.23	0.57	941.65	941.62	<0.005	2.54	2.56	0.79
6	41.30	41.00	−0.73	877.49	879.30	0.21	493.74	493.73	<0.005	7.81	7.86	0.64
7	31.94	32.00	0.19	801.07	800.21	0.11	903.90	903.89	<0.005	5.51	5.50	0.18
8	97.92	98.00	0.08	690.72	690.63	0.01	234.58	234.58	<0.005	6.93	6.92	0.14
9	23.30	23.00	−1.29	606.78	610.67	0.64	595.57	595.52	−0.01	11.47	11.62	1.31
10	73.034	73.00	−0.05	753.37	753.63	0.03	981.51	981.51	<0.005	2.22	2.22	<0.005

**Table 5:** The comparison of the mean and standard deviations of estimated values with the proposed method for 1000 randomly generated datasets with different noise levels.

Noise Level [%]	*w*	*R_s_* [Ω]	*R_p_* [Ω]	*C_p_* [nF]	RMSE Improvement [%]
		220 Ω	1000 Ω	3.3 nF	
0.5%	0.86 ± 0.17	217.17 ± 11.43	1002.41 ± 1.69	3.25 ± 0.08	9.06
1.0%	0.83 ± 0.18	216.04 ± 15.13	1005.17 ± 2.97	3.23 ± 0.10	8.09
5.0%	0.53 ± 0.18	212.73 ± 26.08	1020.16 ± 10.50	3.08 ± 0.16	11.37
10.0%	0.34 ± 0.13	211.47 ± 34.38	1029.51 ± 17.56	2.96 ± 0.20	14.78

**Table 6:** Comparison of estimation results for various numbers of frequency points and noise levels.

*R*_s_ = 330.00 Ω, *R*_p_ = 100.00 Ω and *C*_p_ = 51.34 nF								
*N*	Noise Level: 0.5%				Noise Level: 1%			
	*R*_s_ [Ω]	*R*_p_ [Ω]	*C*_p_ (nF)	RE [%] for *f*_0_	*R*_s_ [Ω]	*R*_p_ [Ω]	*C*_p_ [nF]	RE [%] for *f*_0_
200	330.83	100.25	51.21	<0.005	336.51	100.70	54.50	6.45
150	333.40	100.18	53.72	4.61	334.38	100.48	53.57	4.61
100	330.68	100.21	51.23	<0.005	331.36	100.41	51.13	<0.005
50	334.44	100.08	54.84	6.45	335.44	100.38	54.67	6.45
20	327.32	99.70	49.48	4.07	326.59	99.48	49.59	4.07

**Table 7:** Comparison of estimation results for different approaches.

	[24]	[28]	[29]	MEISP	This Work
*R*s [Ω]	27.42	25.89	28.86	26.58	27.13
*R*_p_ [Ω]	28.61	31.84	26.05	29.07	28.54
*C*_p_ [mF]	3.92	3.55	4.70	3.56	3.93
*RMSE_R_* [Ω]	0.54	1.46	0.80	0.38	0.47
*RMSE_X_* [Ω]	1.01	0.96	3.50	0.90	1.02

## Data Availability

Data will be available upon request to the corresponding author.
